# The Black Art

**Published:** 1838

**Authors:** 


### Art. VI.—The Black Art.

Some time last winter, a colored man was brought to the *Cincinnati Eye Infirmary*, to be relieved, if possible, from the consequence of gonorrhœal opthalmia. He was blind in one eye and nearly so in the other. He stated, that for a long time, he had been a patient in the Philadelphia hospitals, and afterwards of Dr. Williams the "celebrated oculist," with whom he had voyaged from New Orleans to this city. No treatment appeared likely to do good, and we saw no more of him; but in a few weeks were told, that he had commenced the practice of physic! Such, indeed, was the fact, and he is now beyond all dispute the most popular and authoritative practitioner in the "Queen of the West." His elegant gig rolls side by side, with those of our most learned and fashionable M. D's., many of whom he has superseded, in the confidence of their patients; and why should it not be so, for his skill is wonderful.

He is enabled not merely to decide on the present state of the patient, but without information from any source, to rehearse accurately the past, and dip considerably into the future. We could, if required, demonstrate this by several chapters of certificates. His means of diagnosis, are as simple as the results of his skill are astonishing. The patient dips his finger in a glass of water, which forthwith assumes qualities that disclose every thing. When the person is too ill to visit him, the water may be sent in a bottle. If the disease be incurable, the water turns black, as black is the color of mourning. It will be perceived from these facts, that he is a humoralist.

Of the number who have been in daily attendance on this "African doctor," we cannot speak with precision—suffice it to say, that *crowds* are often assembled about his door, waiting anxiously for admittance; that among them are seen persons of wealth and intelligence; that the majority are of the city, but not a few come from parts of the country considerably remote; and that his visiters are of both sexes, with a full proportion of the Fair, who find in the novelty and freshness of being examined and inquired into, by a negro, an irresistable charm.

In his intercourse with his patients, he sustains his character, —there is no flinching or cowering about him—nothing is too high or hard for his genius—he flatters no one—and conceals nothing because it is disagreeable—he is, indeed, a dogmatist as well as a humoralist.

But his skill is by no means limited to the cure of diseases.—One of his patients, who had sought relief for a weakness of the eyes occasioned by grief, for the loss of a husband who had returned to his other wife in Canada, was told by the *doctor* that her husband was absent, and would return in a week, bringing with him another woman and two children! She looked out a whole week for the "dere cratur" in full faith that he would return, because the "doctor" knew nothing of her bereavement from what she or any one else had told him.

Many who have visited him, have been told, precisely, the conversation which had occurred in their families, on the subject of the proposed application.

A woman who was indisposed, was assured by him, that she had cancer uteri. She immediately prepared to return to the eastern states to die with her relatives.

Another laboring under pulmonary consumption, dipped her finger into a tumbler of water. The next day he waited on her, and showed her that the water had turned black, and consequently that she must die. She fell into hystericks.

Another declared, that he had told her every thing that had been done to her in her whole life.

A young lady sent him some water in which she had dipped her finger, he told the bearer, that a man had, also, dipped his finger into it, which was denied, "then," says he, "the young lady is pregnant with a boy child."

These specimens of his skill and discernment, may be of service to such of our non professional readers, as may wish to consult him.

It is said, that this "distinguished individual" was banished not long since from the city of New York, by the combination against him, of a large number of physicians, moved by jealousy of his superior skill; and it is but a few days since, we were told that one of our most celebrated physicians, in attempting to drive him out of town had been dangerously stabbed by him. Delicacy prevents our mentioning the name of this gentleman; but we are happy to state, that we are not likely to lose him.

The good people of Cincinnati, and its vicinity, on both sides of the river, to indemnify themselves for his speculations on *them*, have been speculating on *him*. By some it has been said, that he is a Portugese, and that, as Portugal is the most learned country of Europe, it is no wonder that he knows so much. Others think him a priest of Obi, and that he has drawn his information from the temples of Timbuctoo? Others believe him to be one of the polyglottadæ from the island of Madagascar, as he could speak six languages when he was but five years old. Others, again believe him to be a magician from the East; but the most satisfactory theory of his origin and miraculous skill, is, that he is a lineal descendent of one of the black Eunuchs of ancient Egypt.

We shall leave it with the numerous intelligent ladies and gentlemen of the city, who are under his care, to settle this knotty point in his biography, merely adding, that his statement to us was, that he had been a shop servant to a physician in the state of Maryland. D.

